# Probe-specific mixed-model approach to detect copy number differences using multiplex ligation-dependent probe amplification (MLPA)

**DOI:** 10.1186/1471-2105-9-261

**Published:** 2008-06-04

**Authors:** Juan R González, Josep L Carrasco, Lluís Armengol, Sergi Villatoro, Lluís Jover, Yutaka Yasui, Xavier Estivill

**Affiliations:** 1 Center for research in environmental epidemiology (CREAL), Barcelona, Spain; 2 CIBER en Epidemiología y Salud Pública (CIBERESP), Spain; 3 Biostatistic Unit, Department of Public Health, University of Barcelona, Spain; 4 Genes and Disease Program, Center for Genomic Regulation (CRG), Barcelona, Spain; 5 Department of Public Health Sciences, School of Public Health, University of Alberta, Canada

## Abstract

**Background:**

MLPA method is a potentially useful semi-quantitative method to detect copy number alterations in targeted regions. In this paper, we propose a method for the normalization procedure based on a non-linear mixed-model, as well as a new approach for determining the statistical significance of altered probes based on linear mixed-model. This method establishes a threshold by using different tolerance intervals that accommodates the specific random error variability observed in each test sample.

**Results:**

Through simulation studies we have shown that our proposed method outperforms two existing methods that are based on simple threshold rules or iterative regression. We have illustrated the method using a controlled MLPA assay in which targeted regions are variable in copy number in individuals suffering from different disorders such as Prader-Willi, DiGeorge or Autism showing the best performace.

**Conclusion:**

Using the proposed mixed-model, we are able to determine thresholds to decide whether a region is altered. These threholds are specific for each individual, incorporating experimental variability, resulting in improved sensitivity and specificity as the examples with real data have revealed.

## Background

With the recent technological advances, different genome-wide studies have uncovered an unprecedented number of structural variants in the human genome [1-7], mainly in the form of copy number variations (CNVs). As much as 12% of the human genome has been reported to be variable among different individuals [6]. The important number of genes and other regulatory elements encompassed by those variable regions, makes it very likely for them to have functional and, ultimately, phenotypical consequences [8,9]. In fact, several publications have already correlated the number of copies of different genes with different degrees of disease predisposition [10-12]. Therefore, the identification of DNA copy number is important in understanding genesis and progression of human diseases.

Several techniques and platforms have been developed for genome-wide analysisi of DNA copy number, such as array-based comparative genomic hybridization (aCGH). The goal of the analysis of DNA copy number data using this approach is to partition the whole genome into segments where copy numbers changes between contiguous segments may be present. The ability of aCGH to discern between different number of copies is very limited, thus different kinds of techniques have been developed for targeted, and more precise, analyses of genomic regions. Multiplex Ligation-dependent Probe Amplification (MLPA) [13] is one of the most used technologies among other existing ones such us Quantitative Multiplex PCR of Short Fluorescent (QMPSF) [14,15] or Multiplex Amplifiable Probe Hybridization (MAPH) [16].

**Figure 1:**
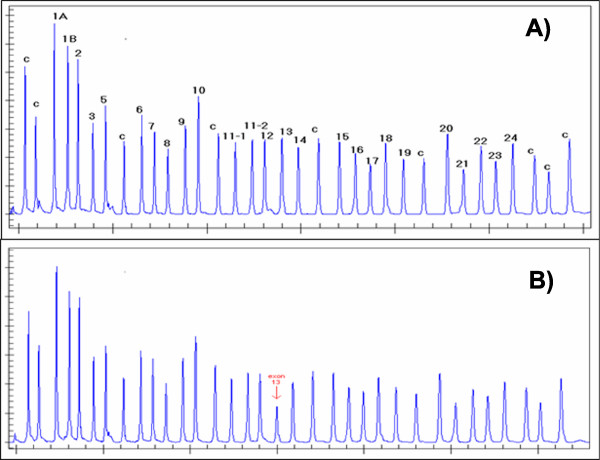
**Example of a deletion in an MLPA assay**. Panels A and B show pherograms corresponding to the electrophoresis of an MLPA assay. In the Y-asix are depicted the intensity signals (peak heights) for each probe that are depicted in the X-axis according to their length (probe size). Peaks marked with a **C **correspond to control probes and peaks numbered from 1 through 24 correspond to region-specific probes. Panel A corresponds to a normal individual, while panel B corresponds to an individual with a deletion at probe #13 as visible by the reduced peak intensity in this pherogram.

MLPA is a recently developed semi-quantitative method that aims to detect copy number alterations at the genomic level (gains or loses) in a test DNA with respect to a control. Due to its low cost, reliability and ease of implementation it has become very popular both as a research and a diagnostic tool. After hybridization, ligation and multiplex amplification of specific probes targeting different genomic regions, the probes are electrophoresed and analyzed using a DNA analyzer [13]. Each specific probe migrates according to its molecular weight and the resulting pherograms show specific peaks that correspond to each probe (Figure 1). Relative dosage information can be obtained after the comparison of peak intensities (height or area) of the different probes between test and control samples.

Due to the variation of PCR efficiencies across probes (due to their different size and nature) and across samples, a normalization method is needed before comparing dosage quotients. This step is crucial because the variation in experimental conditions may lead to differences of measured values between samples, thus hampering the correct interpretation of results. After normalization, which eliminates possible differences introduced during the experimental process, we aim to find biological differences in gene dosage (copy numbers). That is, the normalization tries to minimize the amount of systematic non-biological variation among samples. Different normalization methods have been used for analyzing MLPA data. The most common method divides each intensity by the sum of all intensities in each sample (see, for instance [17] and [18]). Other alternative approaches are based on regression methods that account for the amplification decay of larger probes. One of such regression methods uses internal control probes [19], while the another normalizes intensities based upon the statistically most probable median peak intensities using a median filter [20]. Other authors suggest to normalize peak intensities by using 4 separate peak groups according to increasing frament sizes ot the peaks (see [21] for further details). Finally, a similar approach using the mean intensity of control probes inside a normalization group as the dividing factor was considered in [22].

After an intensity normalization, the next key point is the determination of genes that are significantly altered. As an example, the peak intensity of exon 13 in Figure 1 seems to be lower in patient than in control indicating a deletion. Again, different approaches have been used to find probes that are altered. In this work we are considering ratio- [18,23] and regression-based [19,20] methods. In both approaches, the basis for the analysis is the comparison among normalized peak intensities from patient and control samples using a dosage ratio. In the ratio-based approach, deletions and duplications are given as outliers from the data set after defining a "biological" threshold (i.e. in a diploid genome, two copies exists of each gene). We assume that the simplest, most likely scenario, is an heterozygous gain or a loss of the material, under which ideal ratio values of 1.5 and 0.5 are expected, respectively. In such a simple scenario, and taking into account experimental noise, it is generally accepted in the literature that values below 0.7 and above 1.33 are indicative of loss and gain of a genetic material, respectively [13,18]. The regression-based approach is based on fitting several linear regression models, considering the normalized peak intensity of a given patient as the dependent variable and the normalized peak intensities of the mean control sample as independent variables. Another method based on taking into account the individual noise for each probe (i.e., standard deviation) for all control samples is considered in [24].

Both the ratio- and regression-based methods have general drawbacks. The ratio-based approach always considers the same gain/loss threshold without taking into account the different variability among experiments and among probes. On the other hand, although the regression-based approaches solve this problem, fitting regression models without considering that the independent variable (normalized peak intensity in controls) is also subject to error may lead to wrong conclusions. In addition, the regression method uses confidence intervals for predictions which are built under the normal theory. The normality assumption may be untenable, particularly because of the limited amount of control probes that are typically used in routine experiments (< 10 probes). Finally, none of these procedures considers replicates for each individual, reducing information by averaging the replicate values.

To overcome these difficulties, we propose alternative methods to deal with the normalization and the discovery of alterations when analyzing MLPA data. Our approach is based on both nonlinear (normalization procedure) and linear (to determine alteration) mixed models. The main advantages of our methods are: (1) the variation among individuals is modelled explicitly using a random effect; and (2) the replicates may be easily incorporated in the model. The deletions and duplications will be determined using tolerance intervals. We validate and compare our methods with the existing methods using a controlled experiment. We evaluate the presence of gains and loses in a set of DNAs from normal individuals and individuals affected by known genetic disorders. The probemix we used included probes of single copy number regions located both inside and outside of the genomic disorder regions. A total of 30 samples, including 24 controls and 6 patients, were analyzed blindly.

## Results

### Simulation study

In this section, we investigate the performance of the two existing methods and our proposed approach through a number of simulations. We simulated an hypothetical assay with 45 probes. We also simulated internal control probes (i.e. non-altered) to be used in the iterative regression approach by setting *σ*_*γ *_= 0. Two different scenarios were simulated in order to investigate the performance of REX-MLPA approach given the number of control probes: 10% and 20% of the 45 probes. Finally, the ratios of the rest of probes were simulated as altered (gains or loses) depending on a different percentages: 5%, 10%, 20% and 50% of the probes. These values were generated from a normal distribution under different scenarios varying between probe variability (*σ*_*β *_∈ {0.2, 0.4, 0.6}), probe-test variability (*σ*_*γ *_∈ {0.2, 0.4, 0.6, 1.0, 1.5, 4.0}) and within-probe or random variability (*σ*_*ε *_∈ {0.05, 0.08}). We simulated 3 replicates of each peak intensity for test and control samples. Note that the average of these replicates were considered in the case of using threshold and REX-MLPA approaches. We summarized our simulations by computing the mean number of altered probed simulated in each run, the empirical type-I error and the empirical power to detect gains and loses. These results are based on 1,000 simulations.

Tables 1 and 2 show the results for the case of having 10% of true altered probes when 10% and 20% of probes have been generated as a internal control probes, respectively. The results for the REX-MLPA method were based on building confidence limits at 99% at each iteration. Regarding the empirical type-I error, the REX-MLPA approach usually overestimate the expected 5%, while the threshold method clearly underestimates type-I error since in all simulations the simulated type-I error was closer to 0% (results omitted in the tables). On the other hand, the probe-specific model shows a good performance. As expected, the power of the three methods increases when the probe-test variability increases. That is, the power depends on the size effect of those altered probes. In general, the probe-specific mixed-model outperforms both REX-MLPA and threshold approaches. The threshold approach is clearly underpowered for those cases in which the magnitude of the effect is not large enough. Finally, the same pattern is observed when the random variability is increased (e.g. within-probe variability) but showing lower power. The simulation results for the case of having 5%, 20% and 50% of true altered probes showed similar behaviour and they can be found in the Additional file 1.

**Table 1:** Empirical type-I error and power obtained in 1,000 simulations using the three different approaches: REX (iterative regression), PEMM (probe-specific mixture model) and threshold.

				Type-I error		Power (gains)			Power (loses)		
			
*σ*_*β*_	*σ*_*γ*_	*σ*_*ε*_	${\bar{x}}_{alt}$	REX	PEMM	REX	PEMM	thres	REX	PEMM	thres
0.2	0.2	0.05	3.7	0.073	0.031	0.815	0.745	0.000	0.819	0.751	0.000
0.2	0.2	0.08	3.6	0.071	0.027	0.689	0.546	0.000	0.681	0.541	0.000
0.2	0.4	0.05	3.5	0.081	0.044	0.923	0.891	0.024	0.930	0.908	0.029
0.2	0.4	0.08	3.5	0.079	0.038	0.870	0.811	0.028	0.863	0.814	0.057
0.2	0.6	0.05	3.6	0.081	0.052	0.965	0.955	0.131	0.956	0.956	0.161
0.2	0.6	0.08	3.6	0.086	0.044	0.922	0.889	0.140	0.922	0.900	0.184
0.2	1.0	0.05	3.6	0.081	0.057	0.980	0.983	0.360	0.977	0.972	0.411
0.2	1.0	0.08	3.6	0.085	0.052	0.955	0.954	0.396	0.947	0.939	0.449
0.2	1.5	0.05	3.6	0.081	0.060	0.982	0.978	0.536	0.981	0.983	0.582
0.2	1.5	0.08	3.6	0.084	0.056	0.981	0.986	0.623	0.980	0.975	0.630
0.2	4.0	0.05	3.5	0.078	0.064	0.995	0.993	0.815	0.993	0.994	0.840
0.2	4.0	0.08	3.5	0.079	0.060	0.996	0.993	0.848	0.993	0.995	0.863

0.4	0.2	0.05	3.7	0.079	0.032	0.822	0.729	0.000	0.827	0.737	0.001
0.4	0.2	0.08	3.7	0.070	0.027	0.690	0.522	0.000	0.697	0.537	0.000
0.4	0.4	0.05	3.7	0.081	0.046	0.918	0.897	0.027	0.926	0.905	0.044
0.4	0.4	0.08	3.7	0.078	0.038	0.878	0.807	0.035	0.871	0.825	0.069
0.4	0.6	0.05	3.6	0.080	0.052	0.958	0.949	0.139	0.952	0.942	0.191
0.4	0.6	0.08	3.5	0.083	0.044	0.926	0.903	0.150	0.913	0.881	0.206
0.4	1.0	0.05	3.7	0.080	0.057	0.981	0.982	0.353	0.973	0.977	0.425
0.4	1.0	0.08	3.5	0.081	0.050	0.949	0.953	0.392	0.950	0.934	0.424
0.4	1.5	0.05	3.6	0.083	0.063	0.989	0.985	0.553	0.987	0.989	0.613
0.4	1.5	0.08	3.6	0.080	0.056	0.976	0.973	0.568	0.966	0.967	0.603
0.4	4.0	0.05	3.6	0.085	0.066	0.997	0.996	0.856	0.995	0.995	0.855
0.4	4.0	0.08	3.6	0.086	0.062	0.994	0.991	0.841	0.989	0.990	0.864

0.6	0.2	0.05	3.6	0.075	0.031	0.828	0.740	0.000	0.824	0.741	0.001
0.6	0.2	0.08	3.6	0.071	0.027	0.701	0.529	0.000	0.690	0.520	0.002
0.6	0.4	0.05	3.7	0.082	0.047	0.932	0.916	0.032	0.914	0.902	0.063
0.6	0.4	0.08	3.6	0.077	0.037	0.864	0.815	0.041	0.874	0.835	0.070
0.6	0.6	0.05	3.7	0.082	0.051	0.964	0.949	0.142	0.955	0.944	0.174
0.6	0.6	0.08	3.6	0.078	0.044	0.924	0.885	0.177	0.918	0.887	0.206
0.6	1.0	0.05	3.6	0.081	0.058	0.977	0.970	0.388	0.972	0.974	0.435
0.6	1.0	0.08	3.6	0.080	0.050	0.946	0.935	0.380	0.960	0.945	0.451
0.6	1.5	0.05	3.6	0.087	0.062	0.982	0.981	0.556	0.982	0.985	0.590
0.6	1.5	0.08	3.6	0.080	0.056	0.972	0.972	0.597	0.975	0.973	0.622
0.6	4.0	0.05	3.5	0.080	0.064	0.995	0.995	0.837	0.994	0.993	0.855
0.6	4.0	0.08	3.6	0.082	0.064	0.988	0.991	0.850	0.990	0.990	0.857

These results are for the case of having 20% of probes as a internal control probes (needed for the REX approach) and 10% of probes as a true altered probes. The results are given for different scenarios between probe variability (*σ*_*β*_), probe-test variability (*σ*_*γ*_) and within-probe variability (*σ*_*ε*_). The column ${\bar{x}}_{alt}$ indicates the mean number of simulated altered probes.

**Table 2:** Empirical type-I error and power obtained in 1,000 simulations using the three different approaches: REX (iterative regression), PEMM (probe-specific mixture model) and threshold.

				Type-I error		Power (gains)			Power (loses)		
			
*σ*_*β*_	*σ*_*γ*_	*σ*_*ε*_	${\bar{x}}_{alt}$	REX	PEMM	REX	PEMM	thres	REX	PEMM	thres
0.2	0.2	0.05	3.9	0.044	0.034	0.750	0.765	0.000	0.724	0.747	0.000
0.2	0.2	0.08	3.8	0.043	0.029	0.581	0.584	0.000	0.544	0.564	0.000
0.2	0.4	0.05	4.0	0.048	0.045	0.885	0.919	0.021	0.896	0.922	0.041
0.2	0.4	0.08	3.9	0.049	0.038	0.806	0.827	0.025	0.791	0.820	0.053
0.2	0.6	0.05	4.0	0.054	0.054	0.933	0.954	0.130	0.938	0.957	0.178
0.2	0.6	0.08	4.0	0.051	0.045	0.875	0.903	0.142	0.870	0.888	0.186
0.2	1.0	0.05	3.9	0.053	0.058	0.968	0.976	0.355	0.962	0.978	0.429
0.2	1.0	0.08	4.0	0.045	0.055	0.924	0.954	0.382	0.926	0.954	0.444
0.2	1.5	0.05	4.0	0.047	0.061	0.974	0.983	0.526	0.974	0.986	0.597
0.2	1.5	0.08	4.0	0.052	0.059	0.952	0.971	0.580	0.960	0.974	0.664
0.2	4.0	0.05	3.9	0.053	0.065	0.991	0.993	0.826	0.993	0.997	0.860
0.2	4.0	0.08	4.0	0.049	0.063	0.982	0.990	0.834	0.989	0.993	0.868

0.4	0.2	0.05	4.0	0.044	0.035	0.721	0.754	0.000	0.726	0.752	0.000
0.4	0.2	0.08	3.9	0.045	0.030	0.568	0.544	0.000	0.528	0.550	0.001
0.4	0.4	0.05	3.9	0.048	0.044	0.898	0.912	0.023	0.884	0.919	0.040
0.4	0.4	0.08	4.2	0.050	0.040	0.810	0.823	0.041	0.799	0.830	0.062
0.4	0.6	0.05	4.0	0.047	0.050	0.929	0.945	0.142	0.915	0.940	0.176
0.4	0.6	0.08	4.0	0.046	0.046	0.869	0.895	0.152	0.859	0.894	0.175
0.4	1.0	0.05	4.0	0.051	0.059	0.968	0.983	0.362	0.961	0.976	0.428
0.4	1.0	0.08	3.9	0.050	0.052	0.937	0.956	0.380	0.937	0.951	0.470
0.4	1.5	0.05	4.0	0.049	0.062	0.976	0.981	0.553	0.967	0.978	0.599
0.4	1.5	0.08	4.1	0.050	0.056	0.951	0.969	0.574	0.955	0.971	0.620
0.4	4.0	0.05	3.9	0.047	0.065	0.991	0.995	0.835	0.991	0.997	0.848
0.4	4.0	0.08	4.0	0.050	0.064	0.983	0.988	0.832	0.990	0.997	0.863

0.6	0.2	0.05	4.0	0.045	0.037	0.720	0.748	0.001	0.740	0.775	0.004
0.6	0.2	0.08	4.0	0.044	0.029	0.565	0.584	0.001	0.533	0.572	0.003
0.6	0.4	0.05	4.0	0.051	0.047	0.880	0.896	0.034	0.892	0.915	0.055
0.6	0.4	0.08	4.0	0.047	0.041	0.800	0.824	0.060	0.792	0.818	0.079
0.6	0.6	0.05	4.1	0.051	0.054	0.919	0.938	0.139	0.926	0.947	0.186
0.6	0.6	0.08	3.9	0.047	0.046	0.877	0.893	0.179	0.889	0.924	0.230
0.6	1.0	0.05	4.0	0.050	0.060	0.959	0.976	0.370	0.956	0.971	0.435
0.6	1.0	0.08	4.0	0.048	0.054	0.930	0.947	0.445	0.922	0.948	0.452
0.6	1.5	0.05	4.0	0.049	0.063	0.975	0.983	0.549	0.977	0.985	0.601
0.6	1.5	0.08	3.9	0.049	0.059	0.962	0.973	0.588	0.960	0.965	0.652
0.6	4.0	0.05	3.9	0.053	0.065	0.988	0.993	0.814	0.992	0.994	0.845
0.6	4.0	0.08	3.9	0.055	0.066	0.979	0.987	0.835	0.984	0.995	0.859

These results are for the case of having 10% of probes as a internal control probes (needed for the REX approach) and 10% of probes as a true altered probes. The results are given for different scenarios between probe variability (*σ*_*β*_), probe-test variability (*σ*_*γ*_) and within-probe variability (*σ*_*ε*_). The column ${\bar{x}}_{alt}$ indicates the mean number of simulated altered probes.

### Validation study

In order to validate our proposed method, and compare its performace with those of the existing methods, we have designed a controlled MLPA probemix in which we have included 34 different oligonucleotides, corresponding to 16 different targeted regions with single copy number in normal individuals. Eight of the targeted regions are variable in copy number in individuals suffering from different genomic disorders (see Table 3). A total of 30 DNAs from different individuals were assayed in triplicate in the study. 24 of the DNAs came from unrelated HapMap individuals (i.e. normal general population), 2 DNAs from Prader-Willi syndrome patients (15q11-q13 deletion), 2 DNAs from DiGeorge syndrome patients (22q11 deletion) and 2 DNAs from autistic patients with a duplication in 10q region. After hybridization, ligation and PCR, probes corresponding to the different individuals were electrophoresed and peak intensities were recorded and blindly analyzed using the different methods. MLPA hybridization, ligation and PCR with universal MLPA primers was performed as described elsewhere [13]. PCR products were loaded on a capillar DNA analyzer and electrophoresed. Genescan software was used to analyze the runs and to retrieve peak intensities corresponding to each probe in the different samples.

**Table 3:** MLPA probemix composition

Gene/Region	Genomic location	Probe size	Band	Comments
ENm323	chr6:108,723,531–108,723,590	126	6q21	single copy number region
ENm013	chr7:90,250,124–90,250,183	105	7q21.13	single copy number region
ENm014	chr7:126,866,339–126,866,393	99	7q31.33	single copy number region
RNAseP (RPP30)	chr10:92,621,710–92,621,757	90	10q23.31	single copy number region
ENr222	chr10:92,621,710–92,621,757	147	6q23.2	single copy number region
ENr111	chr13:29,519,123–29,519,182	123	13q12.3	single copy number region
ENr233	chr15:41,662,068–41,662,127	141	15q15.3	single copy number region
ENm313	chr16:61,141,268–61,141,327	114	16q21	single copy number region
ENr213	chr18:24,170,726–24,170,785	132	18q12.1	single copy number region
RP11–71N21	chr10:51,942,608–51,942,682	144	10q11.23	10q duplication
ZWINT	chr10:57,789,521–57,789,580	120	10q21.1	10q duplication
PHYLIP	chr10:60,674,910–60,674,995	138	10q21.1	10q duplication
SNRPN	chr15:22,764,255–22,764,313	111	15q11.2	15q11 deletion
UBEA3A	chr15:23,133,602–23,133,661	129	15q11.2	15q11 deletion
UBE3A	chr15:23,171,946–23,171,999	96	15q11.2	15q11 deletion
HIRA	chr22:17,698,971–17,699,021	93	22q11.21	22q11 deletion

#### Comparison of methods of copy number estimation

Before analyzing the data we illustrate that our proposed method based on a mixed-model can be applied. Supplementary Figure S1 shows the residual error for each individual and probe indicating that the residuals are centered at zero and that the variability does not change with individuals. Table 4 shows the results obtained from the 9 test samples used in the validation study using the threshold, iterative regression and proposed probe-specific mixed-model method. For the two patients with Prader-Willi syndrome, we observed that all methods were able to detect the three deletions in 15q11-q13 region. The same conclusion was observed regarding patients diagnosed with DiGeorge syndrome. Considering patients with Autism, all methods detected gains in the 10q region. Only in one case threshold method had a false negative result and the REX-MLPA method indicated a deletion in the UBE3A, a false positive finding. These results clearly agree with our simulation studies in which we found that iterative regression has a increased type-I error rate and the threshold approach a low power. For instance, the ratio between the second individual diagnosed with Autism and the mean controls was 1.27 at probe named ZWINT, so the threshold method did not indicate that this was a probe altered in this individual. This result is due to the fact that the ratio between the probe-test variability and the random error variability was around 3 (3.09 = $\frac{0.1423}{0.0461}$) and the simulation study showed that in this case the power of threshold approach is very poor. Finally, regarding HapMap individuals who were considered as negative controls, we observed that any method found a false positive result.

**Table 4:** MLPA results from the validation study.

	Prader-Willi						DiGeorge						Autism						HapMap								
				
Gene/Region	#1			#2			#1			#2			#1			#2			#1			#2			#3		
									
ENm323	0	0	0	0	0	0	0	0	0	0	0	0	0	0	0	0	0	0	0	0	0	0	0	0	0	0	0
ENm013	0	0	0	0	0	0	0	0	0	0	0	0	0	0	0	0	0	0	0	0	0	0	0	0	0	0	0
ENm014	0	0	0	0	0	0	0	0	0	0	0	0	0	0	0	0	0	0	0	0	0	0	0	0	0	0	0
RNAseP (RPP30)	0	0	0	0	0	0	0	0	0	0	0	0	0	0	0	0	0	0	0	0	0	0	0	0	0	0	0
ENr222	0	0	0	0	0	0	0	0	0	0	0	0	0	0	0	0	0	0	0	0	0	0	0	0	0	0	0
ENr111	0	0	0	0	0	0	0	0	0	0	0	0	0	0	0	0	0	0	0	0	0	0	0	0	0	0	0
ENr233	0	0	0	0	0	0	0	0	0	0	0	0	0	0	0	0	0	0	0	0	0	0	0	0	0	0	0
ENm313	0	0	0	0	0	0	0	0	0	0	0	0	0	0	0	0	0	0	0	0	0	0	0	0	0	0	0
ENr213	0	0	0	0	0	0	0	0	0	0	0	0	0	0	0	0	0	0	0	0	0	0	0	0	0	0	0
RP11-71N21	0	0	0	0	0	0	0	0	0	0	0	0	1	1	1	1	1	1	0	0	0	0	0	0	0	0	0
ZWINT	0	0	0	0	0	0	0	0	0	0	0	0	1	1	1	**1**	**0**	**1**	0	0	0	0	0	0	0	0	0
PHYLIP	0	0	0	0	0	0	0	0	0	0	0	0	1	1	1	1	1	1	0	0	0	0	0	0	0	0	0
SNRPN	-1	-1	-1	-1	-1	-1	0	0	0	0	0	0	0	0	0	0	0	0	0	0	0	0	0	0	0	0	0
UBEA3A	-1	-1	-1	-1	-1	-1	0	0	0	0	0	0	0	0	0	0	0	0	0	0	0	0	0	0	0	0	0
UBE3A	-1	-1	-1	-1	-1	-1	0	0	0	0	0	0	0	0	0	**0**	**0**	**-1**	0	0	0	0	0	0	0	0	0
HIRA	0	0	0	0	0	0	-1	-1	-1	-1	-1	-1	0	0	0	0	0	0	0	0	0	0	0	0	0	0	0

The three colums for each individual indicates the result obtained using mixed-model, threshold and REX-MLPA approaches, respectively. The code for the results are the following: -1: relative loss, 0:normal, 1:relative gain. Those result where a disagreement between the three methods is observed are in bold face.

## Conclusion

The MLPA method is a potentially useful semi-quantitative method to detect copy number alterations in targeted regions obtained after performing genome-wide screening using comparative methods usually based on genomic hybridization. MLPA is based on comparing peak intensities of the different probes between test and control samples through its ratio. Before determining the statistical significance of dosage ratios, a normalization procedure is needed to control variation of PCR efficiencies across probes. We have proposed a non-linear mixed-model to perform such normalization, and two other existing methods have been discussed. One of them is based on considering the sum of peak intensities in each sample, while another uses internal control probes to fit a regression line which is used as a reference.

So far, the widely used method to detect statistical significance of dosage ratio is to consider as altered those probes that are outside a given threshold. In this paper, we have described a new approach to detect altered probes based on a mixed-model. The threshold is then established by using a tolerance interval which accommodates the specific random error variability observed in each test sample. We have also discussed another approach based on an iterative regression. Through simulation studies and a controlled MLPA assay, we have shown that our proposed method outperforms the existing ones. Another important advantage by comparing our approach and the REX-MLPA method is that our method can be used even when no internal control probes are available in the assay.

A novelty of our algorithm is that it can handle information from replicate experiments. Replicates of MLPA assays are typically not necessary for commercial kits since in most cases more than one probe is used to interrogate the same region and concordance between those probes is considered to provide enough reliability of the existence of a copy number variation. Nevertheless, in cases where a single probe per region is placed in the assay, it is definitely useful and desirable (if not necessary) to have replicates that help minimise possible variations arising during the long MLPA protocol and to ensure reliability of the findings. Thus, having a method that is able to handle such information is very useful and provides extra statistical robustness to the findings.

The use of control probes as references for data normalization is one of the methods recommended by the manufacturer. Commercial and home-made MLPA assays are clearly different in terms of quality of the resulting pherograms, mainly due to the process of producing the specific probes that target regions of interest. While commercial applications produce very high, sharp and neat peaks, home-made assays typically do not perform that well. Nevertheless, in both cases, the use of control probes is always desirable from a statistical point of view and it is so regardless of the number of probes that target a determined loci. In exploratory experiments with MLPA designs targeting extremely variable and polymorphic CNVs, adding control probes whose behaviour is proven unvariable and can help in assessing reliability of single experiments. In such cases, we understand that the inclusion of evenly spaced control probes is a good practice. One key point is to know the number of replicates that should be included in a MLPA assay. Our simulations indicates that 3 replicates was enough to reach a power of 90% keeping a value of alfa around 5% in those combinations where the probe-test variability was over 8 times greater than that of random error (combinations where *σ*_*γ *_= 0.4 and *σ*_*ε *_= 0.05). Thus, if the probe-test versus random error variability ratio was lower, a lesser power would be expected. In that case the researcher could improve the expected power by increasing the number of replicates.

Although our proposed method outperforms other existing ones, it has to be kept in mind that in the case of having probes targeting regions that are highly variable among the population (i.e. in an extreme case where half individuals are normal homozygous and half are homozygously deleted), the tolerance intervals calculated for those probes might collect all existing variability and become very broad, thus making it impossible to distinguish the existence of copy number alteration. Nevertheless, the other existing methods would also show inconsistent copy number calls when analysing such regions. In statistical terms, this means that we should have homogeneity of the random error variance through the probes. This is the reason why a visual inspection of the pherograms as well as manual curation of the results might be the most reliable way to proceed. However, we can easily accomodate cases where the homogeneity of random error variance assumption cannot be assumed by introducing an interaction probe-random error allowing a different random error variance for each probe. In this case, different tolerance bands would be therefore estimated for each probe.

In conclusion, we have proposed a mixed-model which is able to determine thresholds to decide whether a region is altered. These threholds are specific for each individual, incorporating experimental variability, resulting in improved sensitivity and specificity as the examples with real data have revealed. An R language package for the three approaches discussed in this paper and data analyzed will be freely available at our web page [25].

## Methods

To further illustrate the statistical methods described in this section, we are using real data provided by the United Kingdom National Genetics Reference Laboratory of Manchester [26]. In particular we are analyzing a dataset from a breast cancer study (called P002 BRCA1), in which copy number changes of different genes have previously been reported. In this example, 9 control probes and 25 analytical probes were analyzed in a total of 5 controls and 8 different test samples.

### Normalization

Herein, we will focus on methods based on considering the total peak intensities and regression-based approaches. The first and the simplest method of normalization, determines the normalized signal of each probe in a given sample by dividing peak intensity by the sum of all peak intensity of the sample. This method was initially proposed by [13] and is the recommended method by MRC-Holland [27] which provides commercial probes for the copy number analysis of different genome regions for diagnostic purposes. Let us begin by giving some notations to illustrate how this method works. Let *H*_*ipk *_be the peak intensity for the *i*-th individual, *i *= 1, ..., *C*, *C *+ 1, ..., *C *+ *T *(*C *is the number of control samples and *T *denotes the number of test samples), the *p*-th probe, *p *= 1, ..., *P*, and the *k*-th replicate, *k *= 1, ..., *K*. The existing simple method designed for normalizing data considers the average of these replicates, ${\tilde{H}}_{ip}=\sum_{k=1}^{K}H_{ipk}/K$, and divides each measured peak intensity by the sum of all peaks, $S_{i}=\sum_{p=1}^{P}{\tilde{H}}_{ip}$ of that sample, i.e. ${\tilde{Y}}_{ip}={\tilde{H}}_{ip}/S_{i}$.

**Figure 2:**
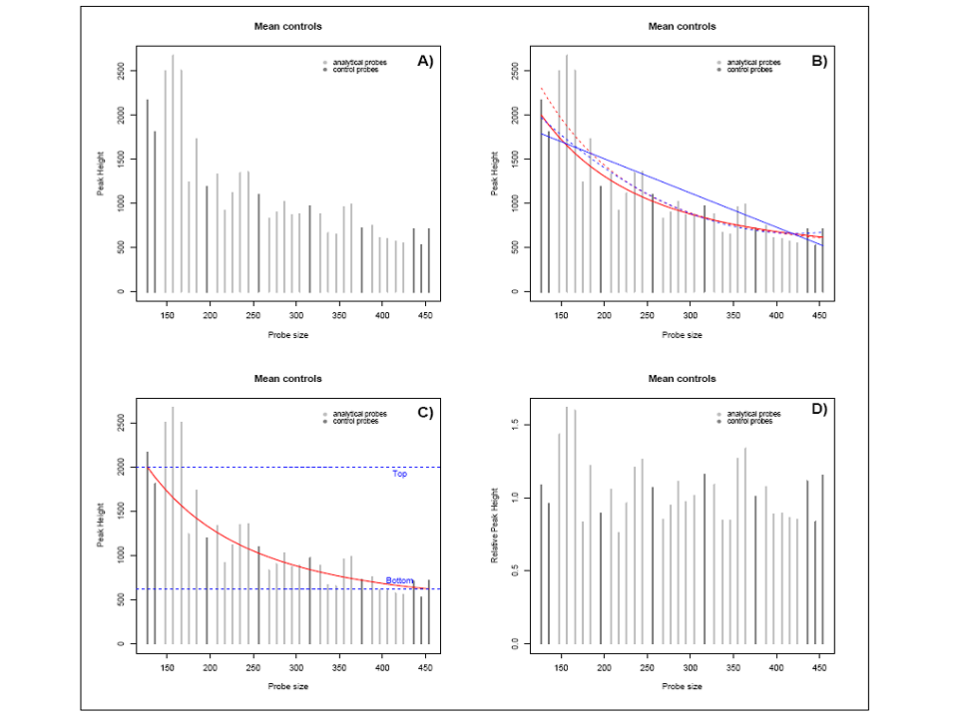
**MLPA normalization procedure**. Panel A shows the peak intensities depending on probe sizes. Panel B shows the regression estimates for different methods: linear regression model using control probes (solid blue line), quadratic model using control probes (dotted blue line), nonlinear mixed model (red lines) using control probes (solid line) or median filter approach (dotted line). Panel C illustrates the parameters involved in the nonlinear mixed model. Panel D shows the size-adjusted normalized peak intensities prepared to compute the dosage quotient. In all panels dark lines represents control probes while light lines are for analytical probes.

In addition to experimental variability, as Figure 1 shows, peak intensity exhibits a negative correlation with probe size. To account for this effect, regression methods may be employed. If internal control probes are provided in the MLPA experiment (dark vertical lines in Figure 2A), a regression-based method using the control probes may also be employed to normalize the data. Different methods have been proposed. The first approach, known as "slope correction" [19], model the negative correlation between probe size and peak intensities using a linear regression model following the formula ${\tilde{H}}_{p_{c}}=\alpha +\beta X_{p_{c}}$, where *X*_*pc *_denotes the size of the *p*_*c*_-th internal control probe for a given individual (Figure 2B solid blue line), or using a quadratic model ${\tilde{H}}_{p_{c}}=\alpha +\beta_{1}X_{p_{c}}+\beta_{2}X_{p_{c}}^{2}$ (Figure 2B, dotted blue line).

Another possibility proposed by [20] is to fit an exponential decay model, ${\tilde{H}}_{p}=\alpha {\text{e}}^{-\beta X_{p}}$. In that case, the authors propose to consider all probes *p *and call this method "population normalization". Notice that in that case *β *encodes the *rate *of descend. [20] argued that the trend of peak intensities varies greatly between samples regarding the control probes. This is the reason why they propose to consider all probes (both analytical and control ones) after applying a median filter approach to remove outliers. However this method has important inconveniences. The smoothed data, obtained after applying the median filter, violates some of the assumptions of nonlinear regression such as that residuals are no longer independent or that errors are not Gaussian, among others [28].

**Figure 3:**
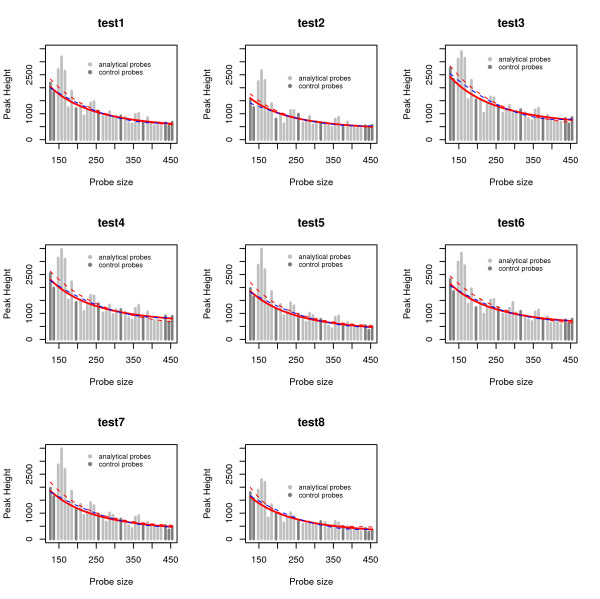
**Regression estimates for the test samples from BRCA1 data set**. Regression estimates for each of the 8 test samples given in the example provided by the NGRL-Manchester called P002 BRCA1. Red lines are estimated using the nonlinear mixed model. The solid lines are estimated using control probes, while dotted lines are obtained after using the median filter approach. The dotted blue lines are showing the regression estimates using quadratic model. These regression lines are then used to normalize the peak intensities.

Neither *slope correction nor population normalization *approaches consider replicates, e.g. they use ${\tilde{H}}_{ip}$ instead of *H*_*ipk*_. In addition, these methods do not model the variability among individuals either. That is, using these methods, the authors allow only *β *to vary among individuals fitting different models for each one. However, the pattern of decay may vary highly between subjects, which is not captured by varying *β *'s only. As an example, the height of the peak intensity for the first control probe (top parameter in Figure 2C) and the asymptotic value for larger probe sizes (bottom parameter in Figure 2C) are very different among test samples, as we observe in Figure 3.

To circumvent these problems, we propose the following nonlinear mixed model to normalize the data which includes replicates and variability among individuals:

(1) $$\begin{array}{c} H_{ipk}=(\varphi_{1i}-\varphi_{2i})\exp[-\frac{1}{\varphi_{3i}}(X_{p}-{\text{off}}_{i})]+\varphi_{2i}+\varepsilon_{ipk},\\ \varphi_{i}=\left[\begin{array}{c} \varphi_{1i}\\ \varphi_{2i}\\ \varphi_{3i} \end{array}\right]=\left[\begin{array}{c} \beta_{1}\\ \beta_{2}\\ \beta_{3} \end{array}\right]+\left[\begin{array}{c} b_{1i}\\ b_{2i}\\ b_{3i} \end{array}\right]=\beta +b_{i},\\ \begin{array}{cc} b_{i}\sim N(0,\Psi ), & \varepsilon_{ijk}\sim N(0,\sigma^{2}), \end{array} \end{array}$$

where *X*_*p *_denotes the size for the *p*-th probe (note that it only depends on the probe since it is the same for each individual and each replicate) *φ*_*i*1 _is the maximum peak intensity for control probes, *φ*_2*i*_, is the asymptotic peak intensity, and *φ*_3*i *_is the reciprocal of the rate decay constant. The term off_*i *_provides a more stable parametrization for the data and corresponds to the average value of the peak size for the first control probe, off_*i *_= $\sum_{k=1}^{K}H_{i1k}$, *i *= 1, ..., *C*, *C *+ 1, ..., *C *+ *T*. The fixed effects, *β *represent the population average of subject-specific parameters, *φ*_*i*_, and the random effects, *b*_*i*_, represents the deviations of the *φ*_*i*_'s from their population average. The random effects are assumed to be independent and the within-group errors *ε*_*ipk *_are assumed to be independent for different *i*, *p *and to be independent of the random effects. The model parameters are fitted by maximizing the restricted log-likelihood (RMLE) of the data using the R library nlme [29].

After estimating this generalized exponential model, the normalization procedure is performed by dividing the peak intensities, *H*_*ipk*_, by the regression estimate, ${\hat{H}}_{ipk}$, obtaining a normalized, size-adjusted peak intensity for every probe, *Y*_*ipk *_= *H*_*ipk*_${\hat{H}}_{ipk}$ (Figure 2D). For the control samples we consider a unique regression line corresponding to the average of all control samples. Nonetheless, for the test samples the normalization is performed individually for each subject.

### Statistical significance of dosage ratios

#### Ratio approach

The normalized peak intensities may be directly compared between control and test samples peak by peak. The first method for determining genes that are altered (gains or loses) is based on a threshold rule. The probes whose dosage ratios are outside of the threshold lines are considered altered. The thresholds are normally 0.7 for deletions (note that 0.5 would correspond to a half reduction in the dosage but this value is considered too stringent by some researchers) and 1.33 for duplications, although other cut-points have been used in other studies [18].

#### Iterative regression

**Figure 4:**
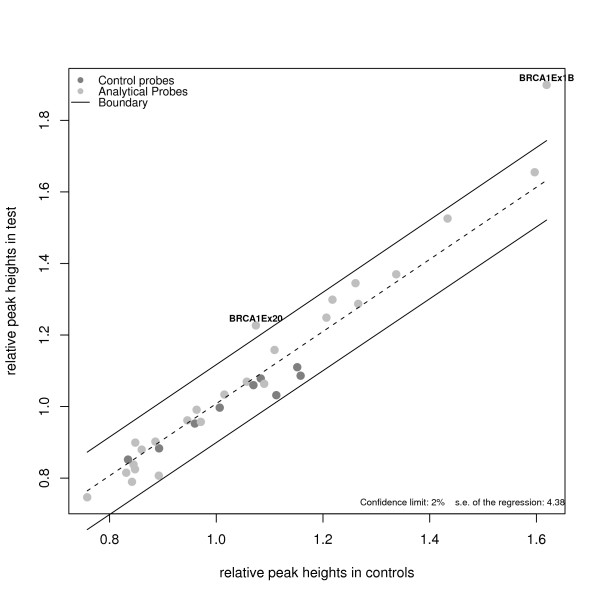
**Normal control comparison procedure using iterative regression**. Plot of normalized peak intensities in controls (calculated as the mean of all controls) against normalized peak intensities in test sample number 1 using iterative regression procedure. Dotted line correspond to linear regression among those probes that are considered non altered after applying the iterative procedure. Solid lines determine upper and lower boundaries which are used to indicate whether or not a given probe is a duplication or a deletion, respectively.

The main disadvantage of defining a threshold is that the specific variability of each experiment is not considered. Some authors have proposed alternative methods using linear regression models. [19] stated that, after a square-root transformation of the data, the plot of normalized peak intensities for test samples versus control samples should be around the diagonal if no dosage imbalances are present; otherwise, probes above or below would correspond to duplications or deletions, respectively. Their proposal was to fit a linear regression without intercept using only control probes and use the standard error of the regression to determine confidence limits for outlier detections. As in the case of normalization procedures, however, a small number of control probes is a limiting factor for adopting this approach since, as the author stated, the standard error of the regression underestimates the normal variability. Thus, [19] proposed an iterative regression algorithm for this problem. Starting with the control probes, the linear regression is fitted. Then, a prediction interval is computed for every probe. If the normalized peak intensity of a non-control probe falls into this interval, the probe is considered to belong to the non-altered population and it is added to the control probes in the next step. After a few iterations, a final model is reached including only those probes that are considered as preserved. In practice, those probes that are not included in this final model are considered to be either deleted or duplicated. The authors call this method Regression-Enhanced MLPA analysis (REX-MLPA). Figure 4 illustrates the confidence limits of the final regression model. [20] proposed a similar approach but starting with all probes and retaining and rejecting points at each iteration with a given level of confidence.

The REX-MLPA approach assumes that the X variable (e.g. squared-root of mean normalized peak intensities in controls) in the linear regression model is measured without error. This assumption is not tenable since this variable corresponds to the normalized peak intensity measured in multiple control samples. Violations of this assumption are a serious problem when trying to accurately predict Y (squared-root of normalized peak intensities in test sample) from X.

#### The probe-specific mixture model

The REX-MLPA approach has the limitations discussed above, specifically, the regression models are fitted without considering that the independent variable (normalized peak intensity in controls) is also subject to error. In addition, confidence intervals for predictions are built assuming the normal theory. This assumption may not hold when only control probes are considered since we typically have no more than 10 probes. Replicates for each individual are also not considered.

Based on these limitations, we propose the following model to compare controls with a given test sample:

(2) $$\begin{array}{c} \log(Y_{tpk})=\mu +\alpha_{t}+\beta_{p}+\gamma_{tp}+\varepsilon_{tpk}\\ \begin{array}{cc} \beta_{p}\sim N(0,\sigma_{\beta }^{2}), & p=1,...,P \end{array}\\ \begin{array}{ccc} \gamma_{tp}\sim N(0,\sigma_{\gamma }^{2}), & t=0,1, & p=1,...,P \end{array}\\ \begin{array}{ccc} \varepsilon_{tpk}\sim N(0,\sigma_{\varepsilon }^{2}), & t=0,1, & \begin{array}{cc} p=1,...,P, & k=1,...,K \end{array} \end{array} \end{array}$$

where *μ *is the mean normalized peak intensity across individuals and probes, *α*_*t *_is a fixed effect representing a different mean for all controls (t = 0) and a given test sample (t = 1), *β*_*p *_is a random effect for the *p*th probe, *γ*_*tp *_is is the probe-test interaction random effect, and *ε*_*tpk *_is a random error variable. The index *p *denotes the probe, and *K *is the number of replicates. We assume that *β*_*p*_, *γ*_*tp*_, and *ε*_*tpk *_are independent, homocedastic, and normally distributed random variables with mean zero. The variances are denoted by $\sigma_{\beta }^{2}$ for the, *β*_*p*_, or the "between-probe" variability, $\sigma_{\gamma }^{2}$ for *γ*_*tp*_, or "probe-test" variability, and $\sigma_{\varepsilon }^{2}$ for the *ε*_*tpk*_, or "within-probe" variability. Considering the assumptions of the proposed model, we need to verify that the within-groups errors are independent and identically normally distributed, with mean zero and variance $\sigma_{\varepsilon }^{2}$, and that they are also independent of the random effect. It can be easily checked using different plots. This assumption makes sense for biologists since probes that behave abnormally in replicate studies or when hybridised onto DNAs that have a different quality are typically removed or redesigned to ensure assay robutness and reliability. The model parameters are fitted by maximizing the restricted log-likelihood (RMLE) using the R library lme (Pinheiro and Bates, 2000).

#### The criterion

For the sake of simplicity, we take control samples as the reference, i.e. *α*_0 _= 0 and *α*_1 _= *α*, and *γ*_0*p *_= 0. Therefore, *α *may be interpreted as an average deviation between test and control samples across all probes, and *γ*_1*p *_as the deviation control-test for the *p*-th probe.

The criterion to determine those probes that are altered for each test sample is based on the observed differences among controls and its differences with controls samples. Conditioned to *p*-th probe, the difference between two control measurements is distributed as

(3) $$\log(Y_{0pl})-\log(Y_{0pk})\sim N(0,2\sigma_{\varepsilon }^{2}).$$

Conversely, the difference between a test, *i'*, and a control sample, conditioned to a given probe, is distributed as

(4) $$\log(Y_{0pk})-\log(Y_{i^{\prime }pk})\sim N(\alpha +\gamma_{i^{\prime }p},2\sigma_{\varepsilon }^{2}),$$

where $\sigma_{\varepsilon }^{2}$ corresponds to the variance of the error defined in equation (2). Then, we consider that a probe is not altered if the mean of the difference between test and control samples is included within a probability interval defined by differences between control samples distribution. Hence, the criterion consists in checking whether the difference between test and control samples is greater than that expected between two control samples. That is, whether the difference is included in the interval

(5) $$(-Z_{1-\kappa /2}\sqrt{2\sigma_{\varepsilon }^{2}},Z_{1-\kappa /2}\sqrt{2\sigma_{\varepsilon }^{2}}),$$

where Z_1-*κ*/2 _is the 1 - *κ*/2 percentile from the standard normal distribution. Hence, 1 - *κ *is the proportion of difference control-control that the difference test-control must exceed to declare that a probe is altered. Nonetheless, when using sample data to estimate such intervals, the uncertainty associated with the estimation estimating them has to be considered. Therefore, we propose to estimate the interval (5) through a tolerance interval over the control-control differences distribution. To build such intervals, it is assumed that $\frac{\nu {\hat{\sigma }}_{e}^{2}}{\sigma_{e}^{2}}\sim \chi_{\nu }^{2}$, where *ν *are the residual degrees of freedom, and ${\hat{\sigma }}_{e}^{2}$ is the REML estimator of $\sigma_{e}^{2}$. Therefore, the tolerance interval that contains (1 - *κ*)% proportion of the control-control differences estimated with a confidence of (1 - *α*)% is

(6) $$\left(-Z_{1-\kappa /2}\sqrt{\frac{\nu {\hat{\sigma }}_{\varepsilon }^{2}}{\chi_{\alpha ,\nu }^{2}}},Z_{1-\kappa /2}\sqrt{\frac{\nu {\hat{\sigma }}_{\varepsilon }^{2}}{\chi_{\alpha ,\nu }^{2}}}\right).$$

**Figure 5:**
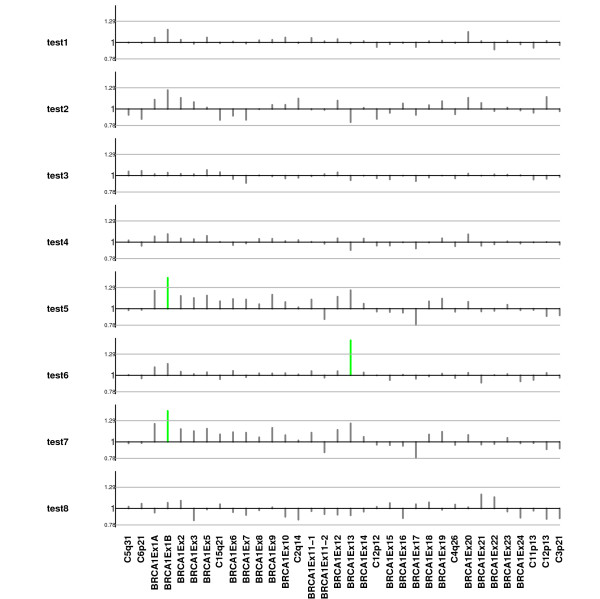
**Normal control comparison procedure using tolerance interval criteria**. Plot of ratio between normalized peak intensities in controls against normalized peak intensities in each of the 8 test samples (vertical lines). Horizontal gray lines indicate lower and upper boundaries obtained using the linear mixed model and tolerance interval criteria. Vertical green lines are indicating that those probes are duplicated for the corresponding probe.

Once the limits of the criterion are established, the mean difference test-control, *δ*_*p *_for each probe, *p*, is estimated using the estimate of *α *+ *γ*_*i'p*_, where *γ*_*p*1 _is estimated through the best linear unbiased predictor (BLUP). Then a probe for a given test sample is considered as altered when *δ*_*p *_does not fall into (6). Figure 5 shows those probes that are imbalanced for each individual considering the threshold obtained using tolerance limits obtained using equation (6). In this example those limits are 0.78 for deletions and 1.29 for duplications.

## Abbreviations

CNV: Copy Number Variant, MLPA: Multiplex Ligation-dependent Probe Amplification, DNA: Deoxyribonucleic acid, aCGH: array-based Comparative Genomic Hybridization, QMPSF: Quantitative Multiplex PCR of Short Fluorescent, PCR: Polymerase Chain Reaction, MAPH: Multiplex Amplifiable Probe Hybridization, BRCA1: Breast Cancer 1, RMLE: Restricted maximum log-likelihood, REX-MLPA: Regression-Enhanced MLPA, BLUP: Best linear unbiased predictor, PEMM: probe-specific mixture model.

## Authors' contributions

JRG and JLC developed the new statistical methods and performed the simulation studies. JRG wrote the R functions and the main text of the manuscript. LA and SV performed the MPLA experiment, interpreted the results and tested the programs functionality. LA, LJ and YY proposed abundant suggestions for improving the implementation of the models and participated in the design and discussion of this study. YY and XE reviewed the paper and revised its framework. All authors have read, and approved the final manuscript.

## Supplementary Material

Additional file 1 mix_model_MLPA.pdf, 173.4K. Click here for file
