# Supplementary material for: Probe-specific mixed-model approach to detect copy number differences using multiplex ligation-dependent probe amplification (MLPA)
Source: BMC Bioinformatics. 2008 Jun 4;9:261. doi: 10.1186/1471-2105-9-261 (PMC2492880; doi:10.1186/1471-2105-9-261)
Supplement: Additional file 1 — mix_model_MLPA.pdf, 173.4K. [file 1471-2105-9-261-S1.pdf]

**Supplementary data of the paper: *Probe-specific mixed-model approach to detect copy number differences using multiplex ligation-dependent probe amplification (MLPA).*** Juan R González, Josep L Carrasco, Lluís Armengol, Sergi Villatoro, Lluís Jover, Yutaka Yasui, Xavier Estivill

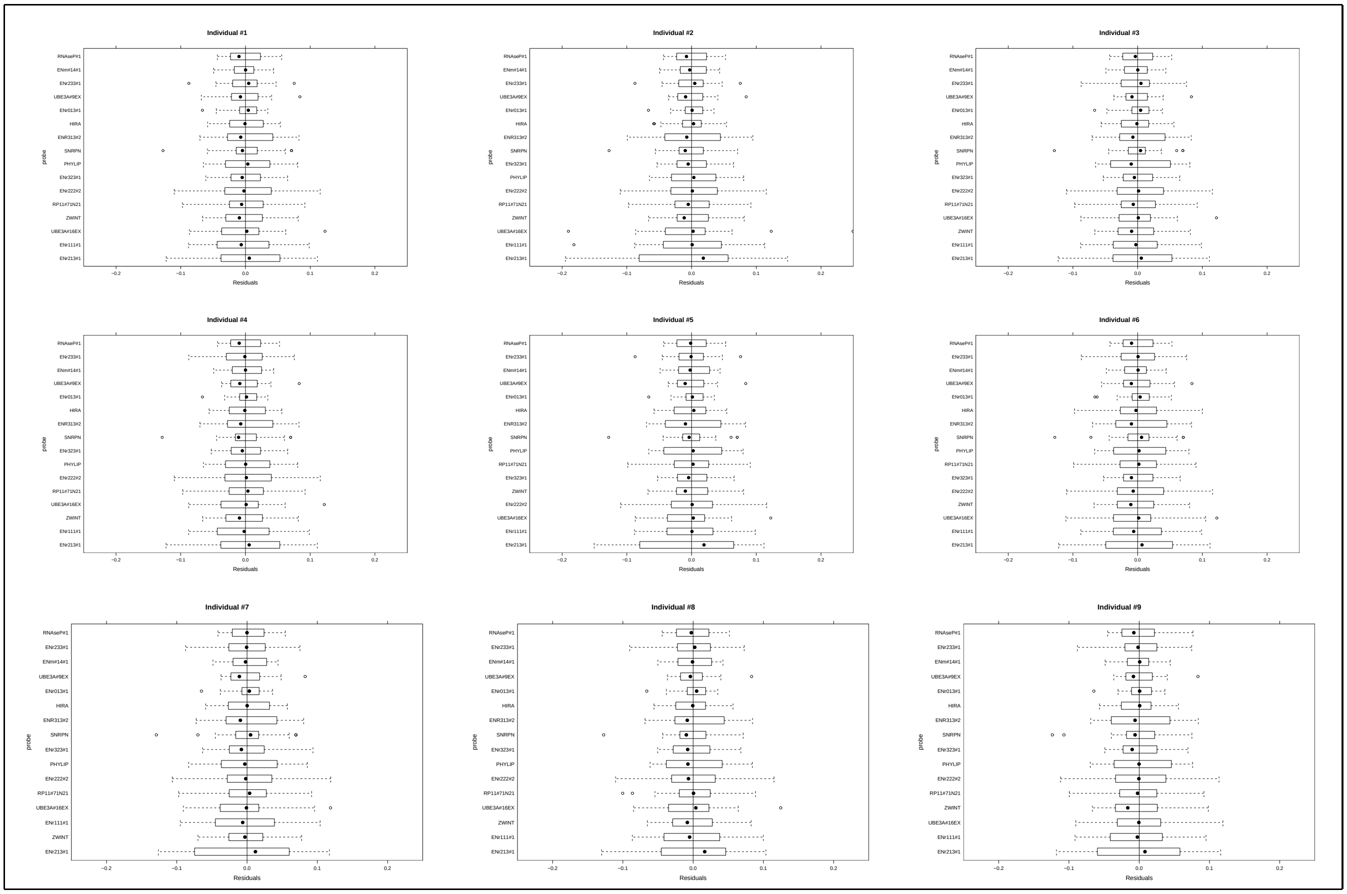


Figure 1: Residual error for each probe after fitting the probe-specific mixed-model.

Table 1: Empirical type-I error and power obtained in 1,000 simulations using the three different approaches: REX (iterative regression), PEMM (probe-specific mixture model) and threshold. These results are for the case of having 20% of probes as a internal control probes (needed for the REX approach) and 50% of probes as a true altered probes. The results are given for different scenarios between probe variability ($\sigma_\beta$), probe-test variability ($\sigma_\gamma$) and within-probe variability ($\sigma_\epsilon$). The column $\bar{x}_{alt}$ indicates the mean number of simulated altered probes.

| $\sigma_\beta$ | $\sigma_\gamma$ | $\sigma_\epsilon$ | $\bar{x}_{alt}$ | REX | PEMM | REX | PEMM | thres | REX | PEMM | thres |
|---|---|---|---|---|---|---|---|---|---|---|---|
| 0.2 | 0.2 | 0.05 | 17.9 | 0.064 | 0.056 | 0.609 | 0.839 | 0.000 | 0.620 | 0.838 | 0.000 |
| 0.2 | 0.2 | 0.08 | 17.7 | 0.059 | 0.048 | 0.396 | 0.697 | 0.000 | 0.392 | 0.695 | 0.000 |
| 0.2 | 0.4 | 0.05 | 18.0 | 0.090 | 0.063 | 0.888 | 0.942 | 0.021 | 0.886 | 0.940 | 0.038 |
| 0.2 | 0.4 | 0.08 | 18.0 | 0.077 | 0.058 | 0.757 | 0.883 | 0.030 | 0.746 | 0.884 | 0.049 |
| 0.2 | 0.6 | 0.05 | 18.0 | 0.093 | 0.065 | 0.944 | 0.962 | 0.122 | 0.946 | 0.962 | 0.164 |
| 0.2 | 0.6 | 0.08 | 18.0 | 0.086 | 0.061 | 0.886 | 0.931 | 0.150 | 0.878 | 0.935 | 0.196 |
| 0.2 | 1.0 | 0.05 | 17.9 | 0.092 | 0.067 | 0.976 | 0.981 | 0.372 | 0.974 | 0.981 | 0.421 |
| 0.2 | 1.0 | 0.08 | 17.9 | 0.092 | 0.064 | 0.953 | 0.967 | 0.393 | 0.948 | 0.966 | 0.446 |
| 0.2 | 1.5 | 0.05 | 18.0 | 0.101 | 0.066 | 0.984 | 0.985 | 0.548 | 0.985 | 0.986 | 0.595 |
| 0.2 | 1.5 | 0.08 | 18.2 | 0.095 | 0.066 | 0.973 | 0.976 | 0.582 | 0.971 | 0.980 | 0.621 |
| 0.2 | 4.0 | 0.05 | 18.1 | 0.107 | 0.068 | 0.995 | 0.996 | 0.832 | 0.995 | 0.995 | 0.845 |
| 0.2 | 4.0 | 0.08 | 18.0 | 0.106 | 0.065 | 0.992 | 0.993 | 0.843 | 0.991 | 0.991 | 0.852 |
| 0.4 | 0.2 | 0.05 | 18.0 | 0.071 | 0.055 | 0.626 | 0.827 | 0.000 | 0.633 | 0.834 | 0.000 |
| 0.4 | 0.2 | 0.08 | 17.8 | 0.048 | 0.049 | 0.360 | 0.700 | 0.000 | 0.358 | 0.698 | 0.001 |
| 0.4 | 0.4 | 0.05 | 18.0 | 0.089 | 0.066 | 0.896 | 0.937 | 0.026 | 0.891 | 0.936 | 0.045 |
| 0.4 | 0.4 | 0.08 | 17.9 | 0.071 | 0.056 | 0.742 | 0.878 | 0.034 | 0.742 | 0.886 | 0.051 |
| 0.4 | 0.6 | 0.05 | 18.0 | 0.097 | 0.067 | 0.946 | 0.963 | 0.132 | 0.947 | 0.959 | 0.169 |
| 0.4 | 0.6 | 0.08 | 18.1 | 0.079 | 0.057 | 0.872 | 0.929 | 0.149 | 0.877 | 0.934 | 0.202 |
| 0.4 | 1.0 | 0.05 | 17.9 | 0.093 | 0.064 | 0.973 | 0.978 | 0.358 | 0.976 | 0.983 | 0.427 |
| 0.4 | 1.0 | 0.08 | 18.0 | 0.094 | 0.060 | 0.952 | 0.963 | 0.401 | 0.947 | 0.961 | 0.444 |
| 0.4 | 1.5 | 0.05 | 18.0 | 0.105 | 0.063 | 0.984 | 0.987 | 0.541 | 0.983 | 0.988 | 0.603 |
| 0.4 | 1.5 | 0.08 | 17.9 | 0.097 | 0.066 | 0.970 | 0.977 | 0.582 | 0.973 | 0.978 | 0.621 |
| 0.4 | 4.0 | 0.05 | 18.0 | 0.099 | 0.071 | 0.995 | 0.995 | 0.825 | 0.995 | 0.996 | 0.845 |
| 0.4 | 4.0 | 0.08 | 17.9 | 0.101 | 0.069 | 0.990 | 0.991 | 0.838 | 0.992 | 0.992 | 0.865 |
| 0.6 | 0.2 | 0.05 | 17.9 | 0.068 | 0.055 | 0.613 | 0.833 | 0.001 | 0.616 | 0.833 | 0.002 |
| 0.6 | 0.2 | 0.08 | 18.0 | 0.048 | 0.048 | 0.358 | 0.690 | 0.003 | 0.342 | 0.693 | 0.003 |
| 0.6 | 0.4 | 0.05 | 18.0 | 0.084 | 0.061 | 0.894 | 0.940 | 0.039 | 0.891 | 0.938 | 0.055 |
| 0.6 | 0.4 | 0.08 | 17.9 | 0.074 | 0.056 | 0.760 | 0.882 | 0.048 | 0.742 | 0.879 | 0.071 |
| 0.6 | 0.6 | 0.05 | 18.1 | 0.093 | 0.064 | 0.951 | 0.966 | 0.149 | 0.946 | 0.960 | 0.186 |
| 0.6 | 0.6 | 0.08 | 17.8 | 0.085 | 0.061 | 0.876 | 0.930 | 0.166 | 0.878 | 0.930 | 0.212 |
| 0.6 | 1.0 | 0.05 | 17.9 | 0.098 | 0.067 | 0.971 | 0.979 | 0.359 | 0.969 | 0.975 | 0.434 |
| 0.6 | 1.0 | 0.08 | 18.0 | 0.089 | 0.062 | 0.952 | 0.964 | 0.411 | 0.950 | 0.963 | 0.460 |
| 0.6 | 1.5 | 0.05 | 17.9 | 0.098 | 0.066 | 0.985 | 0.987 | 0.544 | 0.986 | 0.988 | 0.598 |
| 0.6 | 1.5 | 0.08 | 18.0 | 0.097 | 0.066 | 0.972 | 0.977 | 0.584 | 0.971 | 0.979 | 0.632 |
| 0.6 | 4.0 | 0.05 | 17.8 | 0.096 | 0.068 | 0.995 | 0.996 | 0.838 | 0.994 | 0.996 | 0.848 |
| 0.6 | 4.0 | 0.08 | 18.0 | 0.102 | 0.067 | 0.992 | 0.993 | 0.838 | 0.992 | 0.993 | 0.857 |

Table 2: Empirical type-I error and power obtained in 1,000 simulations using the three different approaches: REX (iterative regression), PEMM (probe-specific mixture model) and threshold. These results are for the case of having 20% of probes as a internal control probes (needed for the REX approach) and 20% of probes as a true altered probes. The results are given for different scenarios between probe variability ($\sigma_\beta$), probe-test variability ($\sigma_\gamma$) and within-probe variability ($\sigma_\epsilon$). The column $\bar{x}_{alt}$ indicates the mean number of simulated altered probes.

| $\sigma_\beta$ | $\sigma_\gamma$ | $\sigma_\epsilon$ | $\bar{x}_{alt}$ | REX | PEMM | REX | PEMM | thres | REX | PEMM | thres |
|---|---|---|---|---|---|---|---|---|---|---|---|
| 0.2 | 0.2 | 0.05 | 7.4 | 0.077 | 0.045 | 0.806 | 0.803 | 0.000 | 0.800 | 0.792 | 0.000 |
| 0.2 | 0.2 | 0.08 | 7.1 | 0.068 | 0.035 | 0.635 | 0.613 | 0.000 | 0.635 | 0.601 | 0.000 |
| 0.2 | 0.4 | 0.05 | 7.2 | 0.083 | 0.054 | 0.923 | 0.918 | 0.020 | 0.911 | 0.920 | 0.038 |
| 0.2 | 0.4 | 0.08 | 7.1 | 0.075 | 0.046 | 0.858 | 0.849 | 0.026 | 0.857 | 0.842 | 0.050 |
| 0.2 | 0.6 | 0.05 | 7.3 | 0.084 | 0.058 | 0.948 | 0.950 | 0.119 | 0.956 | 0.961 | 0.177 |
| 0.2 | 0.6 | 0.08 | 7.1 | 0.080 | 0.052 | 0.919 | 0.918 | 0.148 | 0.919 | 0.927 | 0.193 |
| 0.2 | 1.0 | 0.05 | 7.3 | 0.093 | 0.066 | 0.979 | 0.977 | 0.364 | 0.974 | 0.979 | 0.408 |
| 0.2 | 1.0 | 0.08 | 7.1 | 0.081 | 0.059 | 0.961 | 0.965 | 0.387 | 0.949 | 0.959 | 0.434 |
| 0.2 | 1.5 | 0.05 | 7.2 | 0.084 | 0.066 | 0.985 | 0.991 | 0.538 | 0.980 | 0.984 | 0.583 |
| 0.2 | 1.5 | 0.08 | 7.4 | 0.084 | 0.063 | 0.973 | 0.977 | 0.584 | 0.976 | 0.977 | 0.613 |
| 0.2 | 4.0 | 0.05 | 7.2 | 0.089 | 0.068 | 0.995 | 0.995 | 0.849 | 0.996 | 0.995 | 0.858 |
| 0.2 | 4.0 | 0.08 | 7.2 | 0.092 | 0.068 | 0.990 | 0.992 | 0.847 | 0.991 | 0.991 | 0.854 |
| 0.4 | 0.2 | 0.05 | 7.3 | 0.070 | 0.038 | 0.800 | 0.792 | 0.000 | 0.798 | 0.789 | 0.000 |
| 0.4 | 0.2 | 0.08 | 7.1 | 0.067 | 0.032 | 0.650 | 0.602 | 0.000 | 0.646 | 0.610 | 0.000 |
| 0.4 | 0.4 | 0.05 | 7.2 | 0.085 | 0.055 | 0.925 | 0.922 | 0.032 | 0.928 | 0.927 | 0.043 |
| 0.4 | 0.4 | 0.08 | 7.3 | 0.077 | 0.050 | 0.859 | 0.850 | 0.027 | 0.853 | 0.849 | 0.055 |
| 0.4 | 0.6 | 0.05 | 7.1 | 0.081 | 0.058 | 0.957 | 0.957 | 0.138 | 0.946 | 0.952 | 0.174 |
| 0.4 | 0.6 | 0.08 | 7.3 | 0.084 | 0.054 | 0.916 | 0.919 | 0.160 | 0.924 | 0.923 | 0.193 |
| 0.4 | 1.0 | 0.05 | 7.2 | 0.081 | 0.059 | 0.981 | 0.982 | 0.377 | 0.976 | 0.978 | 0.427 |
| 0.4 | 1.0 | 0.08 | 7.3 | 0.080 | 0.058 | 0.955 | 0.961 | 0.406 | 0.950 | 0.962 | 0.434 |
| 0.4 | 1.5 | 0.05 | 7.1 | 0.080 | 0.065 | 0.986 | 0.983 | 0.545 | 0.979 | 0.984 | 0.599 |
| 0.4 | 1.5 | 0.08 | 7.3 | 0.084 | 0.062 | 0.975 | 0.979 | 0.591 | 0.974 | 0.973 | 0.625 |
| 0.4 | 4.0 | 0.05 | 7.2 | 0.086 | 0.067 | 0.997 | 0.998 | 0.828 | 0.993 | 0.994 | 0.836 |
| 0.4 | 4.0 | 0.08 | 7.2 | 0.087 | 0.066 | 0.991 | 0.993 | 0.847 | 0.989 | 0.991 | 0.859 |
| 0.6 | 0.2 | 0.05 | 7.3 | 0.073 | 0.041 | 0.803 | 0.778 | 0.001 | 0.806 | 0.792 | 0.001 |
| 0.6 | 0.2 | 0.08 | 7.2 | 0.066 | 0.035 | 0.651 | 0.608 | 0.002 | 0.651 | 0.596 | 0.004 |
| 0.6 | 0.4 | 0.05 | 7.3 | 0.082 | 0.053 | 0.928 | 0.928 | 0.040 | 0.931 | 0.929 | 0.052 |
| 0.6 | 0.4 | 0.08 | 7.1 | 0.078 | 0.045 | 0.868 | 0.867 | 0.041 | 0.866 | 0.846 | 0.076 |
| 0.6 | 0.6 | 0.05 | 7.1 | 0.085 | 0.059 | 0.950 | 0.955 | 0.135 | 0.948 | 0.955 | 0.175 |
| 0.6 | 0.6 | 0.08 | 7.2 | 0.081 | 0.052 | 0.919 | 0.925 | 0.167 | 0.917 | 0.907 | 0.206 |
| 0.6 | 1.0 | 0.05 | 7.2 | 0.084 | 0.065 | 0.973 | 0.978 | 0.369 | 0.978 | 0.981 | 0.444 |
| 0.6 | 1.0 | 0.08 | 7.3 | 0.087 | 0.060 | 0.959 | 0.967 | 0.402 | 0.952 | 0.953 | 0.449 |
| 0.6 | 1.5 | 0.05 | 7.3 | 0.086 | 0.064 | 0.984 | 0.984 | 0.559 | 0.984 | 0.987 | 0.580 |
| 0.6 | 1.5 | 0.08 | 7.1 | 0.090 | 0.067 | 0.969 | 0.976 | 0.568 | 0.973 | 0.973 | 0.631 |
| 0.6 | 4.0 | 0.05 | 7.1 | 0.084 | 0.067 | 0.995 | 0.995 | 0.828 | 0.991 | 0.993 | 0.842 |
| 0.6 | 4.0 | 0.08 | 7.2 | 0.088 | 0.066 | 0.990 | 0.993 | 0.831 | 0.992 | 0.996 | 0.862 |

Table 3: Empirical type-I error and power obtained in 1,000 simulations using the three different approaches: REX (iterative regression), PEMM (probe-specific mixture model) and threshold. These results are for the case of having 20% of probes as a internal control probes (needed for the REX approach) and 5% of probes as a true altered probes. The results are given for different scenarios between probe variability ($\sigma_\beta$), probe-test variability ($\sigma_\gamma$) and within-probe variability ($\sigma_\epsilon$). The column $\bar{x}_{alt}$ indicates the mean number of simulated altered probes.

| $\sigma_\beta$ | $\sigma_\gamma$ | $\sigma_\epsilon$ | $\bar{x}_{alt}$ | REX | PEMM | REX | PEMM | thres | REX | PEMM | thres |
|---|---|---|---|---|---|---|---|---|---|---|---|
| 0.2 | 0.2 | 0.05 | 1.8 | 0.080 | 0.025 | 0.846 | 0.700 | 0.000 | 0.835 | 0.703 | 0.000 |
| 0.2 | 0.2 | 0.08 | 1.8 | 0.080 | 0.022 | 0.720 | 0.495 | 0.000 | 0.716 | 0.498 | 0.000 |
| 0.2 | 0.4 | 0.05 | 1.8 | 0.082 | 0.032 | 0.941 | 0.906 | 0.018 | 0.941 | 0.883 | 0.045 |
| 0.2 | 0.4 | 0.08 | 1.8 | 0.078 | 0.026 | 0.884 | 0.778 | 0.033 | 0.855 | 0.762 | 0.040 |
| 0.2 | 0.6 | 0.05 | 1.9 | 0.081 | 0.040 | 0.946 | 0.924 | 0.113 | 0.953 | 0.919 | 0.173 |
| 0.2 | 0.6 | 0.08 | 1.8 | 0.080 | 0.030 | 0.924 | 0.853 | 0.136 | 0.931 | 0.870 | 0.179 |
| 0.2 | 1.0 | 0.05 | 1.8 | 0.077 | 0.046 | 0.971 | 0.957 | 0.367 | 0.982 | 0.971 | 0.447 |
| 0.2 | 1.0 | 0.08 | 1.7 | 0.081 | 0.038 | 0.972 | 0.951 | 0.406 | 0.966 | 0.943 | 0.447 |
| 0.2 | 1.5 | 0.05 | 1.8 | 0.077 | 0.051 | 0.977 | 0.978 | 0.572 | 0.984 | 0.981 | 0.573 |
| 0.2 | 1.5 | 0.08 | 1.8 | 0.080 | 0.044 | 0.970 | 0.953 | 0.551 | 0.968 | 0.955 | 0.604 |
| 0.2 | 4.0 | 0.05 | 1.8 | 0.082 | 0.055 | 0.999 | 0.994 | 0.815 | 0.997 | 0.997 | 0.838 |
| 0.2 | 4.0 | 0.08 | 1.8 | 0.078 | 0.050 | 0.984 | 0.985 | 0.821 | 0.985 | 0.977 | 0.823 |
| 0.4 | 0.2 | 0.05 | 1.7 | 0.076 | 0.024 | 0.844 | 0.706 | 0.000 | 0.846 | 0.701 | 0.000 |
| 0.4 | 0.2 | 0.08 | 1.8 | 0.077 | 0.021 | 0.712 | 0.495 | 0.000 | 0.716 | 0.508 | 0.000 |
| 0.4 | 0.4 | 0.05 | 1.8 | 0.078 | 0.033 | 0.932 | 0.883 | 0.023 | 0.933 | 0.879 | 0.039 |
| 0.4 | 0.4 | 0.08 | 1.8 | 0.079 | 0.027 | 0.877 | 0.773 | 0.044 | 0.870 | 0.749 | 0.051 |
| 0.4 | 0.6 | 0.05 | 1.9 | 0.080 | 0.040 | 0.952 | 0.932 | 0.145 | 0.961 | 0.931 | 0.176 |
| 0.4 | 0.6 | 0.08 | 1.9 | 0.081 | 0.034 | 0.912 | 0.852 | 0.157 | 0.925 | 0.866 | 0.188 |
| 0.4 | 1.0 | 0.05 | 1.8 | 0.083 | 0.046 | 0.984 | 0.962 | 0.390 | 0.983 | 0.979 | 0.458 |
| 0.4 | 1.0 | 0.08 | 1.8 | 0.078 | 0.039 | 0.964 | 0.946 | 0.385 | 0.950 | 0.928 | 0.424 |
| 0.4 | 1.5 | 0.05 | 1.8 | 0.080 | 0.052 | 0.981 | 0.975 | 0.543 | 0.993 | 0.988 | 0.599 |
| 0.4 | 1.5 | 0.08 | 1.8 | 0.077 | 0.045 | 0.974 | 0.953 | 0.563 | 0.965 | 0.949 | 0.647 |
| 0.4 | 4.0 | 0.05 | 1.8 | 0.083 | 0.056 | 0.997 | 0.999 | 0.830 | 0.997 | 0.993 | 0.832 |
| 0.4 | 4.0 | 0.08 | 1.8 | 0.080 | 0.053 | 0.993 | 0.988 | 0.831 | 0.992 | 0.989 | 0.860 |
| 0.6 | 0.2 | 0.05 | 1.7 | 0.078 | 0.025 | 0.830 | 0.688 | 0.000 | 0.842 | 0.708 | 0.003 |
| 0.6 | 0.2 | 0.08 | 1.7 | 0.072 | 0.021 | 0.733 | 0.514 | 0.000 | 0.716 | 0.518 | 0.002 |
| 0.6 | 0.4 | 0.05 | 1.8 | 0.080 | 0.035 | 0.921 | 0.872 | 0.037 | 0.917 | 0.866 | 0.053 |
| 0.6 | 0.4 | 0.08 | 1.8 | 0.078 | 0.026 | 0.879 | 0.772 | 0.060 | 0.882 | 0.773 | 0.086 |
| 0.6 | 0.6 | 0.05 | 1.8 | 0.083 | 0.040 | 0.959 | 0.935 | 0.123 | 0.957 | 0.948 | 0.192 |
| 0.6 | 0.6 | 0.08 | 1.8 | 0.078 | 0.032 | 0.909 | 0.862 | 0.169 | 0.917 | 0.873 | 0.211 |
| 0.6 | 1.0 | 0.05 | 1.8 | 0.081 | 0.046 | 0.974 | 0.958 | 0.356 | 0.973 | 0.968 | 0.400 |
| 0.6 | 1.0 | 0.08 | 1.9 | 0.083 | 0.040 | 0.963 | 0.939 | 0.383 | 0.965 | 0.933 | 0.472 |
| 0.6 | 1.5 | 0.05 | 1.8 | 0.075 | 0.053 | 0.984 | 0.984 | 0.549 | 0.986 | 0.979 | 0.614 |
| 0.6 | 1.5 | 0.08 | 1.8 | 0.080 | 0.045 | 0.977 | 0.968 | 0.600 | 0.983 | 0.971 | 0.643 |
| 0.6 | 4.0 | 0.05 | 1.8 | 0.083 | 0.057 | 0.994 | 0.996 | 0.833 | 0.996 | 0.993 | 0.876 |
| 0.6 | 4.0 | 0.08 | 1.9 | 0.082 | 0.055 | 0.991 | 0.995 | 0.864 | 0.993 | 0.990 | 0.853 |

Table 4: Empirical type-I error and power obtained in 1,000 simulations using the three different approaches: REX (iterative regression), PEMM (probe-specific mixture model) and threshold. These results are for the case of having 10% of probes as a internal control probes (needed for the REX approach) and 50% of probes as a true altered probes. The results are given for different scenarios between probe variability ($\sigma_\beta$), probe-test variability ($\sigma_\gamma$) and within-probe variability ($\sigma_\epsilon$). The column $\bar{x}_{alt}$ indicates the mean number of simulated altered probes.

| $\sigma_\beta$ | $\sigma_\gamma$ | $\sigma_\epsilon$ | $\bar{x}_{alt}$ | REX | PEMM | REX | PEMM | thres | REX | PEMM | thres |
|---|---|---|---|---|---|---|---|---|---|---|---|
| 0.2 | 0.2 | 0.05 | 20.0 | 0.031 | 0.055 | 0.380 | 0.834 | 0.000 | 0.374 | 0.842 | 0.000 |
| 0.2 | 0.2 | 0.08 | 19.8 | 0.024 | 0.048 | 0.174 | 0.705 | 0.000 | 0.184 | 0.707 | 0.000 |
| 0.2 | 0.4 | 0.05 | 19.9 | 0.048 | 0.062 | 0.789 | 0.936 | 0.020 | 0.780 | 0.937 | 0.034 |
| 0.2 | 0.4 | 0.08 | 20.0 | 0.037 | 0.061 | 0.523 | 0.890 | 0.028 | 0.519 | 0.888 | 0.049 |
| 0.2 | 0.6 | 0.05 | 20.1 | 0.050 | 0.065 | 0.894 | 0.966 | 0.123 | 0.892 | 0.961 | 0.159 |
| 0.2 | 0.6 | 0.08 | 20.1 | 0.042 | 0.061 | 0.743 | 0.930 | 0.146 | 0.735 | 0.935 | 0.197 |
| 0.2 | 1.0 | 0.05 | 19.8 | 0.055 | 0.063 | 0.953 | 0.979 | 0.367 | 0.949 | 0.978 | 0.407 |
| 0.2 | 1.0 | 0.08 | 20.0 | 0.049 | 0.063 | 0.893 | 0.965 | 0.395 | 0.891 | 0.964 | 0.451 |
| 0.2 | 1.5 | 0.05 | 20.1 | 0.056 | 0.064 | 0.972 | 0.989 | 0.555 | 0.971 | 0.987 | 0.595 |
| 0.2 | 1.5 | 0.08 | 20.0 | 0.053 | 0.061 | 0.948 | 0.978 | 0.580 | 0.944 | 0.977 | 0.631 |
| 0.2 | 4.0 | 0.05 | 19.9 | 0.056 | 0.065 | 0.990 | 0.997 | 0.823 | 0.990 | 0.995 | 0.843 |
| 0.2 | 4.0 | 0.08 | 20.1 | 0.059 | 0.066 | 0.984 | 0.993 | 0.840 | 0.986 | 0.993 | 0.866 |
| 0.4 | 0.2 | 0.05 | 20.0 | 0.036 | 0.059 | 0.388 | 0.837 | 0.000 | 0.394 | 0.834 | 0.000 |
| 0.4 | 0.2 | 0.08 | 19.9 | 0.022 | 0.049 | 0.175 | 0.707 | 0.000 | 0.172 | 0.700 | 0.001 |
| 0.4 | 0.4 | 0.05 | 20.1 | 0.042 | 0.062 | 0.773 | 0.936 | 0.024 | 0.769 | 0.933 | 0.044 |
| 0.4 | 0.4 | 0.08 | 20.2 | 0.037 | 0.059 | 0.506 | 0.888 | 0.035 | 0.503 | 0.881 | 0.057 |
| 0.4 | 0.6 | 0.05 | 19.8 | 0.049 | 0.062 | 0.893 | 0.962 | 0.134 | 0.888 | 0.961 | 0.167 |
| 0.4 | 0.6 | 0.08 | 20.1 | 0.043 | 0.062 | 0.735 | 0.935 | 0.154 | 0.736 | 0.934 | 0.198 |
| 0.4 | 1.0 | 0.05 | 20.1 | 0.056 | 0.069 | 0.950 | 0.979 | 0.362 | 0.947 | 0.980 | 0.419 |
| 0.4 | 1.0 | 0.08 | 20.1 | 0.050 | 0.064 | 0.906 | 0.964 | 0.391 | 0.898 | 0.961 | 0.452 |
| 0.4 | 1.5 | 0.05 | 20.0 | 0.053 | 0.067 | 0.969 | 0.985 | 0.546 | 0.968 | 0.987 | 0.593 |
| 0.4 | 1.5 | 0.08 | 20.0 | 0.053 | 0.067 | 0.945 | 0.982 | 0.577 | 0.946 | 0.978 | 0.630 |
| 0.4 | 4.0 | 0.05 | 20.0 | 0.058 | 0.067 | 0.990 | 0.995 | 0.826 | 0.990 | 0.995 | 0.840 |
| 0.4 | 4.0 | 0.08 | 20.1 | 0.053 | 0.066 | 0.984 | 0.993 | 0.839 | 0.983 | 0.993 | 0.863 |
| 0.6 | 0.2 | 0.05 | 20.0 | 0.035 | 0.054 | 0.386 | 0.844 | 0.002 | 0.386 | 0.837 | 0.002 |
| 0.6 | 0.2 | 0.08 | 19.9 | 0.022 | 0.049 | 0.175 | 0.699 | 0.003 | 0.166 | 0.700 | 0.003 |
| 0.6 | 0.4 | 0.05 | 20.0 | 0.047 | 0.066 | 0.757 | 0.937 | 0.039 | 0.769 | 0.938 | 0.057 |
| 0.6 | 0.4 | 0.08 | 20.2 | 0.042 | 0.059 | 0.516 | 0.884 | 0.049 | 0.518 | 0.884 | 0.073 |
| 0.6 | 0.6 | 0.05 | 20.1 | 0.048 | 0.067 | 0.889 | 0.961 | 0.143 | 0.880 | 0.960 | 0.183 |
| 0.6 | 0.6 | 0.08 | 20.2 | 0.043 | 0.062 | 0.739 | 0.934 | 0.168 | 0.739 | 0.933 | 0.219 |
| 0.6 | 1.0 | 0.05 | 19.8 | 0.055 | 0.064 | 0.952 | 0.979 | 0.369 | 0.947 | 0.979 | 0.433 |
| 0.6 | 1.0 | 0.08 | 19.9 | 0.049 | 0.068 | 0.900 | 0.967 | 0.399 | 0.890 | 0.962 | 0.448 |
| 0.6 | 1.5 | 0.05 | 19.9 | 0.053 | 0.068 | 0.971 | 0.987 | 0.558 | 0.971 | 0.987 | 0.595 |
| 0.6 | 1.5 | 0.08 | 20.0 | 0.050 | 0.065 | 0.940 | 0.976 | 0.585 | 0.942 | 0.974 | 0.615 |
| 0.6 | 4.0 | 0.05 | 20.0 | 0.061 | 0.071 | 0.990 | 0.995 | 0.834 | 0.988 | 0.994 | 0.845 |
| 0.6 | 4.0 | 0.08 | 20.1 | 0.056 | 0.066 | 0.982 | 0.992 | 0.843 | 0.984 | 0.994 | 0.861 |

Table 5: Empirical type-I error and power obtained in 1,000 simulations using the three different approaches: REX (iterative regression), PEMM (probe-specific mixture model) and threshold. These results are for the case of having 10% of probes as a internal control probes (needed for the REX approach) and 20% of probes as a true altered probes. The results are given for different scenarios between probe variability ($\sigma_\beta$), probe-test variability ($\sigma_\gamma$) and within-probe variability ($\sigma_\epsilon$). The column $\bar{x}_{alt}$ indicates the mean number of simulated altered probes.

| $\sigma_\beta$ | $\sigma_\gamma$ | $\sigma_\epsilon$ | $\bar{x}_{alt}$ | REX | PEMM | REX | PEMM | thres | REX | PEMM | thres |
|---|---|---|---|---|---|---|---|---|---|---|---|
| 0.2 | 0.2 | 0.05 | 8.0 | 0.044 | 0.044 | 0.692 | 0.804 | 0.000 | 0.706 | 0.818 | 0.000 |
| 0.2 | 0.2 | 0.08 | 7.9 | 0.034 | 0.035 | 0.477 | 0.631 | 0.000 | 0.473 | 0.612 | 0.000 |
| 0.2 | 0.4 | 0.05 | 7.9 | 0.049 | 0.057 | 0.870 | 0.928 | 0.020 | 0.872 | 0.931 | 0.040 |
| 0.2 | 0.4 | 0.08 | 8.0 | 0.043 | 0.045 | 0.776 | 0.855 | 0.027 | 0.753 | 0.856 | 0.048 |
| 0.2 | 0.6 | 0.05 | 8.1 | 0.050 | 0.061 | 0.927 | 0.962 | 0.129 | 0.929 | 0.963 | 0.172 |
| 0.2 | 0.6 | 0.08 | 8.0 | 0.049 | 0.057 | 0.861 | 0.926 | 0.151 | 0.856 | 0.916 | 0.177 |
| 0.2 | 1.0 | 0.05 | 7.9 | 0.052 | 0.065 | 0.956 | 0.978 | 0.339 | 0.961 | 0.981 | 0.407 |
| 0.2 | 1.0 | 0.08 | 7.9 | 0.049 | 0.061 | 0.929 | 0.965 | 0.419 | 0.936 | 0.962 | 0.460 |
| 0.2 | 1.5 | 0.05 | 7.9 | 0.051 | 0.064 | 0.972 | 0.986 | 0.567 | 0.968 | 0.986 | 0.608 |
| 0.2 | 1.5 | 0.08 | 8.1 | 0.051 | 0.061 | 0.960 | 0.977 | 0.579 | 0.959 | 0.981 | 0.618 |
| 0.2 | 4.0 | 0.05 | 8.1 | 0.049 | 0.067 | 0.987 | 0.995 | 0.829 | 0.992 | 0.997 | 0.847 |
| 0.2 | 4.0 | 0.08 | 7.9 | 0.052 | 0.065 | 0.984 | 0.989 | 0.838 | 0.987 | 0.994 | 0.876 |
| 0.4 | 0.2 | 0.05 | 8.0 | 0.040 | 0.045 | 0.690 | 0.808 | 0.000 | 0.684 | 0.801 | 0.000 |
| 0.4 | 0.2 | 0.08 | 8.1 | 0.034 | 0.037 | 0.479 | 0.642 | 0.001 | 0.471 | 0.624 | 0.001 |
| 0.4 | 0.4 | 0.05 | 8.0 | 0.049 | 0.056 | 0.871 | 0.922 | 0.025 | 0.878 | 0.929 | 0.042 |
| 0.4 | 0.4 | 0.08 | 8.0 | 0.041 | 0.046 | 0.753 | 0.846 | 0.030 | 0.765 | 0.845 | 0.054 |
| 0.4 | 0.6 | 0.05 | 8.0 | 0.050 | 0.062 | 0.925 | 0.963 | 0.145 | 0.924 | 0.958 | 0.171 |
| 0.4 | 0.6 | 0.08 | 8.0 | 0.045 | 0.056 | 0.861 | 0.922 | 0.154 | 0.853 | 0.919 | 0.199 |
| 0.4 | 1.0 | 0.05 | 7.9 | 0.054 | 0.064 | 0.963 | 0.982 | 0.384 | 0.959 | 0.982 | 0.417 |
| 0.4 | 1.0 | 0.08 | 8.0 | 0.048 | 0.062 | 0.928 | 0.960 | 0.395 | 0.931 | 0.961 | 0.455 |
| 0.4 | 1.5 | 0.05 | 8.0 | 0.053 | 0.065 | 0.981 | 0.989 | 0.571 | 0.972 | 0.987 | 0.598 |
| 0.4 | 1.5 | 0.08 | 8.0 | 0.050 | 0.062 | 0.957 | 0.977 | 0.597 | 0.962 | 0.976 | 0.632 |
| 0.4 | 4.0 | 0.05 | 7.9 | 0.055 | 0.069 | 0.991 | 0.996 | 0.836 | 0.989 | 0.992 | 0.838 |
| 0.4 | 4.0 | 0.08 | 8.0 | 0.050 | 0.067 | 0.986 | 0.997 | 0.850 | 0.985 | 0.994 | 0.864 |
| 0.6 | 0.2 | 0.05 | 7.9 | 0.042 | 0.046 | 0.680 | 0.799 | 0.001 | 0.670 | 0.787 | 0.002 |
| 0.6 | 0.2 | 0.08 | 8.0 | 0.035 | 0.036 | 0.470 | 0.633 | 0.005 | 0.474 | 0.633 | 0.006 |
| 0.6 | 0.4 | 0.05 | 8.0 | 0.045 | 0.055 | 0.871 | 0.923 | 0.035 | 0.868 | 0.924 | 0.056 |
| 0.6 | 0.4 | 0.08 | 8.0 | 0.037 | 0.049 | 0.772 | 0.865 | 0.050 | 0.755 | 0.852 | 0.078 |
| 0.6 | 0.6 | 0.05 | 8.2 | 0.053 | 0.063 | 0.925 | 0.961 | 0.139 | 0.931 | 0.960 | 0.191 |
| 0.6 | 0.6 | 0.08 | 7.9 | 0.047 | 0.057 | 0.858 | 0.913 | 0.162 | 0.865 | 0.925 | 0.207 |
| 0.6 | 1.0 | 0.05 | 8.0 | 0.050 | 0.063 | 0.957 | 0.979 | 0.366 | 0.970 | 0.983 | 0.439 |
| 0.6 | 1.0 | 0.08 | 8.0 | 0.052 | 0.061 | 0.929 | 0.960 | 0.401 | 0.924 | 0.961 | 0.446 |
| 0.6 | 1.5 | 0.05 | 8.1 | 0.052 | 0.067 | 0.973 | 0.985 | 0.555 | 0.974 | 0.987 | 0.602 |
| 0.6 | 1.5 | 0.08 | 8.0 | 0.050 | 0.063 | 0.952 | 0.976 | 0.567 | 0.957 | 0.976 | 0.631 |
| 0.6 | 4.0 | 0.05 | 8.0 | 0.051 | 0.066 | 0.989 | 0.994 | 0.824 | 0.992 | 0.996 | 0.855 |
| 0.6 | 4.0 | 0.08 | 8.1 | 0.051 | 0.068 | 0.983 | 0.993 | 0.843 | 0.985 | 0.994 | 0.862 |

Table 6: Empirical type-I error and power obtained in 1,000 simulations using the three different approaches: REX (iterative regression), PEMM (probe-specific mixture model) and threshold. These results are for the case of having 10% of probes as a internal control probes (needed for the REX approach) and 5% of probes as a true altered probes. The results are given for different scenarios between probe variability ($\sigma_\beta$), probe-test variability ($\sigma_\gamma$) and within-probe variability ($\sigma_\epsilon$). The column $\bar{x}_{alt}$ indicates the mean number of simulated altered probes.

| $\sigma_\beta$ | $\sigma_\gamma$ | $\sigma_\epsilon$ | $\bar{x}_{alt}$ | REX | PEMM | REX | PEMM | thres | REX | PEMM | thres |
|---|---|---|---|---|---|---|---|---|---|---|---|
| 0.2 | 0.2 | 0.05 | 2.0 | 0.048 | 0.027 | 0.756 | 0.733 | 0.000 | 0.751 | 0.730 | 0.000 |
| 0.2 | 0.2 | 0.08 | 2.0 | 0.044 | 0.023 | 0.573 | 0.518 | 0.000 | 0.599 | 0.552 | 0.000 |
| 0.2 | 0.4 | 0.05 | 2.0 | 0.052 | 0.037 | 0.881 | 0.874 | 0.023 | 0.895 | 0.892 | 0.049 |
| 0.2 | 0.4 | 0.08 | 1.9 | 0.046 | 0.028 | 0.794 | 0.781 | 0.030 | 0.785 | 0.765 | 0.047 |
| 0.2 | 0.6 | 0.05 | 2.0 | 0.049 | 0.043 | 0.940 | 0.946 | 0.141 | 0.935 | 0.931 | 0.151 |
| 0.2 | 0.6 | 0.08 | 2.0 | 0.047 | 0.036 | 0.882 | 0.893 | 0.147 | 0.859 | 0.855 | 0.175 |
| 0.2 | 1.0 | 0.05 | 2.1 | 0.047 | 0.047 | 0.970 | 0.974 | 0.383 | 0.964 | 0.973 | 0.410 |
| 0.2 | 1.0 | 0.08 | 2.0 | 0.053 | 0.045 | 0.936 | 0.942 | 0.419 | 0.918 | 0.931 | 0.443 |
| 0.2 | 1.5 | 0.05 | 2.0 | 0.052 | 0.052 | 0.980 | 0.982 | 0.546 | 0.982 | 0.988 | 0.592 |
| 0.2 | 1.5 | 0.08 | 1.9 | 0.052 | 0.045 | 0.958 | 0.967 | 0.590 | 0.958 | 0.958 | 0.597 |
| 0.2 | 4.0 | 0.05 | 2.0 | 0.050 | 0.057 | 0.994 | 0.996 | 0.826 | 0.993 | 0.994 | 0.849 |
| 0.2 | 4.0 | 0.08 | 1.9 | 0.048 | 0.057 | 0.987 | 0.986 | 0.818 | 0.985 | 0.985 | 0.846 |
| 0.4 | 0.2 | 0.05 | 2.0 | 0.045 | 0.026 | 0.782 | 0.719 | 0.000 | 0.753 | 0.706 | 0.000 |
| 0.4 | 0.2 | 0.08 | 2.0 | 0.048 | 0.024 | 0.598 | 0.549 | 0.000 | 0.607 | 0.548 | 0.002 |
| 0.4 | 0.4 | 0.05 | 2.0 | 0.046 | 0.038 | 0.897 | 0.886 | 0.031 | 0.882 | 0.888 | 0.054 |
| 0.4 | 0.4 | 0.08 | 2.0 | 0.047 | 0.032 | 0.812 | 0.824 | 0.038 | 0.811 | 0.789 | 0.065 |
| 0.4 | 0.6 | 0.05 | 2.1 | 0.048 | 0.041 | 0.936 | 0.936 | 0.133 | 0.935 | 0.937 | 0.193 |
| 0.4 | 0.6 | 0.08 | 2.0 | 0.049 | 0.034 | 0.873 | 0.864 | 0.170 | 0.863 | 0.866 | 0.184 |
| 0.4 | 1.0 | 0.05 | 2.0 | 0.048 | 0.050 | 0.955 | 0.959 | 0.362 | 0.962 | 0.967 | 0.432 |
| 0.4 | 1.0 | 0.08 | 2.0 | 0.050 | 0.042 | 0.924 | 0.928 | 0.403 | 0.943 | 0.947 | 0.462 |
| 0.4 | 1.5 | 0.05 | 2.0 | 0.048 | 0.052 | 0.979 | 0.980 | 0.537 | 0.974 | 0.981 | 0.574 |
| 0.4 | 1.5 | 0.08 | 2.0 | 0.051 | 0.046 | 0.962 | 0.963 | 0.576 | 0.958 | 0.966 | 0.617 |
| 0.4 | 4.0 | 0.05 | 1.9 | 0.048 | 0.057 | 0.992 | 0.994 | 0.835 | 0.992 | 0.994 | 0.831 |
| 0.4 | 4.0 | 0.08 | 2.0 | 0.049 | 0.055 | 0.987 | 0.988 | 0.841 | 0.982 | 0.993 | 0.860 |
| 0.6 | 0.2 | 0.05 | 2.0 | 0.046 | 0.028 | 0.735 | 0.729 | 0.001 | 0.752 | 0.710 | 0.002 |
| 0.6 | 0.2 | 0.08 | 2.0 | 0.041 | 0.023 | 0.597 | 0.505 | 0.003 | 0.593 | 0.522 | 0.003 |
| 0.6 | 0.4 | 0.05 | 2.0 | 0.049 | 0.037 | 0.887 | 0.884 | 0.027 | 0.910 | 0.899 | 0.068 |
| 0.6 | 0.4 | 0.08 | 2.0 | 0.047 | 0.029 | 0.810 | 0.801 | 0.047 | 0.798 | 0.787 | 0.074 |
| 0.6 | 0.6 | 0.05 | 2.0 | 0.052 | 0.043 | 0.923 | 0.923 | 0.160 | 0.927 | 0.946 | 0.175 |
| 0.6 | 0.6 | 0.08 | 1.9 | 0.047 | 0.033 | 0.867 | 0.871 | 0.140 | 0.878 | 0.881 | 0.215 |
| 0.6 | 1.0 | 0.05 | 2.0 | 0.052 | 0.049 | 0.962 | 0.958 | 0.376 | 0.964 | 0.968 | 0.435 |
| 0.6 | 1.0 | 0.08 | 1.9 | 0.048 | 0.042 | 0.927 | 0.921 | 0.380 | 0.957 | 0.949 | 0.482 |
| 0.6 | 1.5 | 0.05 | 2.0 | 0.048 | 0.052 | 0.973 | 0.973 | 0.514 | 0.970 | 0.984 | 0.583 |
| 0.6 | 1.5 | 0.08 | 2.0 | 0.050 | 0.048 | 0.973 | 0.975 | 0.574 | 0.972 | 0.967 | 0.645 |
| 0.6 | 4.0 | 0.05 | 2.0 | 0.050 | 0.058 | 0.991 | 0.994 | 0.829 | 0.991 | 0.992 | 0.857 |
| 0.6 | 4.0 | 0.08 | 2.1 | 0.049 | 0.058 | 0.986 | 0.983 | 0.845 | 0.986 | 0.991 | 0.855 |
